# Supplementary material for: Emerging Fusarium Mycotoxins Fusaproliferin, Beauvericin, Enniatins, and Moniliformin in Serbian Maize
Source: Toxins (Basel). 2019 Jun 19;11(6):357. doi: 10.3390/toxins11060357 (PMC6628450; doi:10.3390/toxins11060357)
Supplement: Supplementary file 1 [file toxins-11-00357-s001.pdf]

Supplementary Materials: Emerging Fusarium Mycotoxins Fusaproliferin, Beauvericin, Enniatins, and Moniliformin in Serbian Maize

Igor Jajić, Tatjana Dudaš, Saša Krstović, Rudolf Krska, Michael Sulyok, Ferenc Bagi, Zagorka Savić, Darko Guljaš and Aleksandra Stankov

Normality test:

[1] "Moniliformin"

Shapiro-Wilk normality test

data: df[[i]]

W = 0.71536, p-value = 3.833e-15

[1] "Beauvericin"

Shapiro-Wilk normality test

data: df[[i]]

W = 0.45344, p-value < 2.2e-16

[1] "Fusaproliferin"

Shapiro-Wilk normality test

data: df[[i]]

W = 0.12177, p-value < 2.2e-16

[1] "April.Temp"

Shapiro-Wilk normality test

data: df[[i]]

W = 0.83133, p-value = 2.184e-11

[1] "May.Temp"

Shapiro-Wilk normality test

data: df[[i]]

W = 0.81032, p-value = 3.536e-12

[1] "June.Temp"

Shapiro-Wilk normality test

data: df[[i]]

W = 0.78115, p-value = 3.503e-13

[1] "July.Temp"

Shapiro-Wilk normality test

data: df[[i]]

W = 0.87256, p-value = 1.279e-09

[1] "August.Temp"

Shapiro-Wilk normality test

data: df[[i]]

W = 0.67654, p-value = 3.76e-16

[1] "September.Temp"

Shapiro-Wilk normality test

data: df[[i]]

W = 0.81324, p-value = 4.517e-12

[1] "April.Precip"

Shapiro-Wilk normality test

data: df[[i]]

W = 0.77426, p-value = 2.096e-13

[1] "May.Precip"

Shapiro-Wilk normality test

data: df[[i]]

W = 0.76691, p-value = 1.224e-13

[1] "June.Precip"

Shapiro-Wilk normality test

data: df[[i]]

W = 0.7955, p-value = 1.062e-12

[1] "July.Precip"

Shapiro-Wilk normality test

data: df[[i]]

W = 0.76561, p-value = 1.115e-13

[1] "August.Precip"

Shapiro-Wilk normality test

data: df[[i]]

W = 0.85182, p-value = 1.504e-10

[1] "September.Precip"

Shapiro-Wilk normality test

data: df[[i]]

W = 0.84275, p-value = 6.272e-11

[1] "Temp"

Shapiro-Wilk normality test

data: df[[i]]

W = 0.78317, p-value = 4.082e-13

[1] "Precip"

Shapiro-Wilk normality test

data: df[[i]]

W = 0.86504, p-value = 5.74e-10

Spearman Correlation

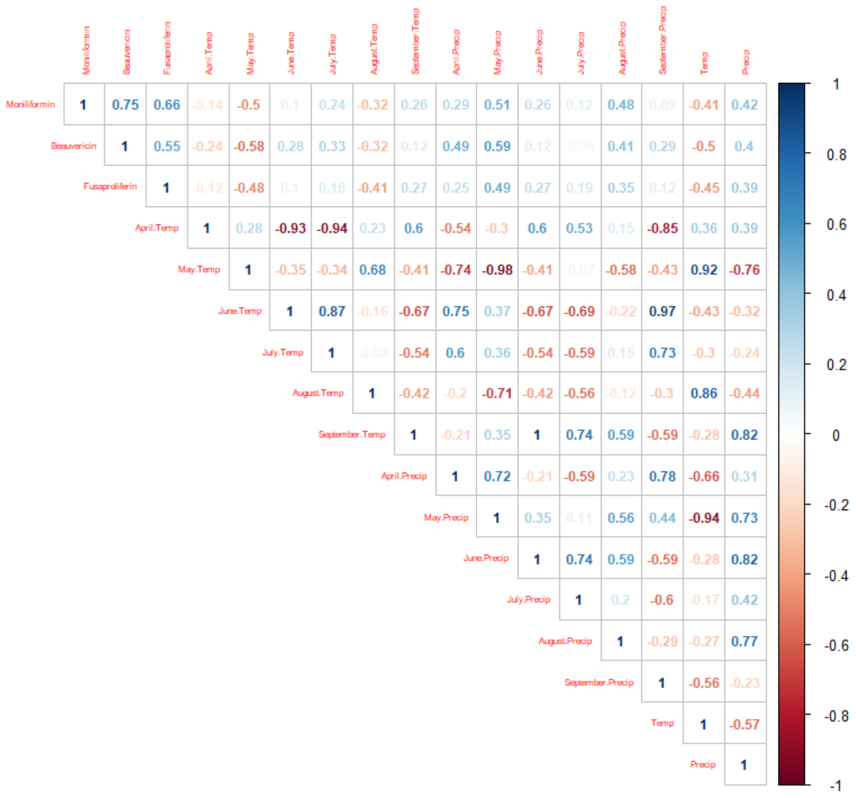

Figure S1. Spearman Correlation Results (*r*).

Kruskal-Wallis rank sum test

>kruskal.test(analysis_data2$Moniliformin~analysis_data2$Region)

Kruskal-Wallis rank sum test

data: analysis_data2$Moniliformin by analysis_data2$Region

Kruskal-Wallis chi-squared = 5.4866, df = 1, p-value = 0.01916

>kruskal.test(analysis_data2$Moniliformin~analysis_data2$Year)

Kruskal-Wallis rank sum test

data: analysis_data2$Moniliformin by analysis_data2$Year

Kruskal-Wallis chi-squared = 33.225, df = 2, p-value = 6.101e-08

>kruskal.test(analysis_data2$Moniliformin~analysis_data2$Temp)

Kruskal-Wallis rank sum test

data: analysis_data2$Moniliformin by analysis_data2$Temp

Kruskal-Wallis chi-squared = 45.42, df = 5, p-value = 1.192e-08

>kruskal.test(analysis_data2$Moniliformin~analysis_data2$Precip)

Kruskal-Wallis rank sum test

data: analysis_data2$Moniliformin by analysis_data2$Precip

Kruskal-Wallis chi-squared = 45.42, df = 5, p-value = 1.192e-08

>kruskal.test(analysis_data2$Fusaproliferin~analysis_data2$Region)

Kruskal-Wallis rank sum test

data: analysis_data2$Fusaproliferin by analysis_data2$Region

Kruskal-Wallis chi-squared = 1.6736, df = 1, p-value = 0.1958

>kruskal.test(analysis_data2$Fusaproliferin~analysis_data2$Year)

Kruskal-Wallis rank sum test

data: analysis_data2$Fusaproliferin by analysis_data2$Year

Kruskal-Wallis chi-squared = 35.254, df = 2, p-value = 2.211e-08

>kruskal.test(analysis_data2$Fusaproliferin~analysis_data2$Temp)

Kruskal-Wallis rank sum test

data: analysis_data2$Fusaproliferin by analysis_data2$Temp

Kruskal-Wallis chi-squared = 38.228, df = 5, p-value = 3.396e-07

>kruskal.test(analysis_data2$Fusaproliferin~analysis_data2$Precip)

Kruskal-Wallis rank sum test

data: analysis_data2$Fusaproliferin by analysis_data2$Precip

Kruskal-Wallis chi-squared = 38.228, df = 5, p-value = 3.396e-07

>kruskal.test(analysis_data2$Beauvericin~analysis_data2$Region)

Kruskal-Wallis rank sum test

data: analysis_data2$Beauvericin by analysis_data2$Region

Kruskal-Wallis chi-squared = 14.166, df = 1, p-value = 0.0001673

>kruskal.test(analysis_data2$Beauvericin~analysis_data2$Year)

Kruskal-Wallis rank sum test

data: analysis_data2$Beauvericin by analysis_data2$Year

Kruskal-Wallis chi-squared = 42.572, df = 2, p-value = 5.697e-10

>kruskal.test(analysis_data2$Beauvericin~analysis_data2$Temp)

Kruskal-Wallis rank sum test

data: analysis_data2$Beauvericin by analysis_data2$Temp

Kruskal-Wallis chi-squared = 53.931, df = 5, p-value = 2.165e-10

>kruskal.test(analysis_data2$Beauvericin~analysis_data2$Precip)

Kruskal-Wallis rank sum test

data: analysis_data2$Beauvericin by analysis_data2$Precip

Kruskal-Wallis chi-squared = 53.931, df = 5, p-value = 2.165e-10

Stepwise regression with backward steps

> full.model <- lm(Moniliformin ~., data = df)

> # Stepwise regression model

>step.model<- stepAIC(full.model, direction = "backward", trace = FALSE)

>summary(step.model)

Call:

lm(formula = Moniliformin ~ Beauvericin + May.Temp + June.Temp +

July.Temp + August.Temp, data = df)

Residuals:

Min 1Q Median 3Q Max

-438.96 -108.10 -88.63 66.95 1413.07

Coefficients:

Estimate Std. Error t value Pr(>|t|)

(Intercept) -4152.033 2374.424 -1.749 0.08264 .

Beauvericin 3.109 1.366 2.276 0.02445 *

May.Temp 148.220 63.831 2.322 0.02174 *

June.Temp -282.054 137.055 -2.058 0.04153 *

July.Temp 529.457 126.171 4.196 4.91e-05 ***

August.Temp -183.888 62.182 -2.957 0.00367 **

Signif. codes: 0 '***' 0.001 '**' 0.01 '*' 0.05 '.' 0.1 ' ' 1

Residual standard error: 300.8 on 134 degrees of freedom

Multiple R-squared: 0.2555, Adjusted R-squared: 0.2278

F-statistic: 9.199 on 5 and 134 DF, p-value: 1.512e-07

> full.model <- lm(Fusaproliferin ~., data = df)

> # Stepwise regression model

>step.model<- stepAIC(full.model, direction = "backward", trace = FALSE)

>summary(step.model)

Call:

lm(formula = Fusaproliferin ~ 1, data = df)

Residuals:

Min 1Q Median 3Q Max

-522 -522 -522 -243 38088

Coefficients:

Estimate Std. Error t value Pr(>|t|)

(Intercept) 522.4 278.4 1.877 0.0627 .

Signif. codes: 0 '***' 0.001 '**' 0.01 '*' 0.05 '.' 0.1 ' ' 1

Residual standard error: 3294 on 139 degrees of freedom

>full.model<- lm(Beauvericin ~., data = df)

> # Stepwise regression model

>step.model<- stepAIC(full.model, direction = "backward", trace = FALSE)

>summary(step.model)

Call:

```
lm(formula = Beauvericin ~ Moniliformin + May.Temp, data = df)

Residuals:

Min      1Q  Median      3Q     Max

-23.204  -8.008  -5.297   1.140 116.738

Coefficients:

            Estimate Std. Error t value Pr(>|t|)

(Intercept)  53.171322  19.505027   2.726  0.00725 **

Moniliformin  0.011551   0.004858   2.378  0.01879 *

May.Temp     -2.685720   1.067909  -2.515  0.01306 *

---

Signif. codes:  0 ‘***’ 0.001 ‘**’ 0.01 ‘*’ 0.05 ‘.’ 0.1 ‘ ’ 1

Residual standard error: 18.58 on 137 degrees of freedom

Multiple R-squared:  0.1139,	Adjusted R-squared:  0.101

F-statistic: 8.806 on 2 and 137 DF,  p-value: 0.0002525
```
